# Supplementary material for: Confirmation of a hyperendemic focus of porcine cysticercosis in Northern Uganda: Prevalence and risk factor analysis
Source: PLoS Negl Trop Dis. 2025 Aug 5;19(8):e0013313. doi: 10.1371/journal.pntd.0013313 (PMC12380272; doi:10.1371/journal.pntd.0013313)
Supplement: S1 Table — (DOCX) [file pntd.0013313.s001.docx]

**S1: Variables collected for the Analysis**

| **Variable Name** | **Full name** | **Levels** |
| --- | --- | --- |
| Household_ | Unique Household ID | 714 |
| District | The district | 1=Kitgum, 2=Lamwo, 3=Agago, 4=Pader |
| Sub County | The sub-county within the district | 24 |
| Village | The Village | 165 (Although the target was 144, some villages had less than 5 eligible pig-rearing households) |
| Gender | The gender of the respondent | 1=Male, 2=Female |
| Age | The age of the respondent | Age in years |
| Respondent | The relation to the household head | 1=House_head, 2=Other |
| Education | The education of the respondent | 0=Primary school or below, 1=Secondary school or equivalent, 2=Higher education |
| Pig roaming | Are pigs allowed to roam sometimes? | 0=No, 1=Yes |
| Borehole water | Borehole water as a source of water | 0=No, 1=Yes |
| Home slaughtering | Slaughter pigs at home | 0=No, 1=Yes |
| Eating pork out | Eat pork outside home | 0=No, 1=Yes |
| Toilet Compound | Is a toilet seen in the compound? | 0=No, 1=Yes |
| Child relieve | How are child faeces disposed of in the latrine? | 0=No, 1=Yes |
| Deworm Family | Deworming family members | 0=Never, 1=Sometimes |
| Number Of Pigs | number of pigs in the household | range from 1 to 46 |
| Pigid | The unique tag number for the sampled pigs | range from 1 upwards |
| Sex of pig | sex of the pig sampled | 1=Male, 2=Female |
| Breed of pig | Pig breed | 1=Local, 2=Non local |
| Age of pig | The age of the pig | Age in months (3 -120 months) |
| Cyst_Status. | The result of ELISA | 0=Negative, 1=Positive |
| Slope_Mean | The slope at the household location | |
| Distance to River | Distance to the river |  |
| Dist_Hc_Hu | Distance to the Health Centre |  |
| Landuse_Co | Land use cover |  |
| Mean_Ndvi_ | Mean NDVI |  |
| Avg_Rai | Average rainfall |  |
| Dist_Roads | Distance to the roads |  |
