# Supplementary material for: Confirmation of a hyperendemic focus of porcine cysticercosis in Northern Uganda: Prevalence and risk factor analysis
Source: PLoS Negl Trop Dis. 2025 Aug 5;19(8):e0013313. doi: 10.1371/journal.pntd.0013313 (PMC12380272; doi:10.1371/journal.pntd.0013313)
Supplement: S2 Table — (DOCX) [file pntd.0013313.s002.docx]

**S2: Sources and Data types of environmental variables**

| **Variable** | **Source** | **Data type** |  |
| --- | --- | --- | --- |
| Landcover | Classification of Sentinel image in QGIS | Raster |  |
| Hospitals | https://data.humdata.org/dataset/hotosm_uga_health_facilities | Vector |  |
| Rivers | DIVA-GIS (https://diva-gis.org/data.html) | Vector |  |
| Normalized difference vegetation index (NDVI | Generated from Google Earth Engine | Raster |  |
| Rainfall | CHIRPS (<https://data.chc.ucsb.edu/products/CHIRPS-2.0/>) | Raster |  |
| Altitude | DIVA-GIS (https://diva-gis.org/data.html) | Raster |  |
| Slope | Generated from Altitude data using QGIS | Raster |  |
