# Supplementary material for: Confirmation of a hyperendemic focus of porcine cysticercosis in Northern Uganda: Prevalence and risk factor analysis
Source: PLoS Negl Trop Dis. 2025 Aug 5;19(8):e0013313. doi: 10.1371/journal.pntd.0013313 (PMC12380272; doi:10.1371/journal.pntd.0013313)
Supplement: S3 Table — (DOCX) [file pntd.0013313.s003.docx]

**S3 Table: Summary of the number of villages, households and pigs sampled**

| **District** | **Agago** | **Kitgum** | **Lamwo** | **Pader** | **Overall** |
| --- | --- | --- | --- | --- | --- |
| Villages | 39 | 38 | 48 | 39 | 164 |
| Households | 179 | 180 | 178 | 177 | 714 |
| Pigs | 270 | 251 | 265 | 263 | 1049 |
