# Supplementary material for: Confirmation of a hyperendemic focus of porcine cysticercosis in Northern Uganda: Prevalence and risk factor analysis
Source: PLoS Negl Trop Dis. 2025 Aug 5;19(8):e0013313. doi: 10.1371/journal.pntd.0013313 (PMC12380272; doi:10.1371/journal.pntd.0013313)
Supplement: S4 Table — (DOCX) [file pntd.0013313.s004.docx]

| **S4 Table: Key pig husbandry practices** | | | | | | | |
| --- | --- | --- | --- | --- | --- | --- | --- |
| Pig husbandry practice | Agago (n=179) | Kitgum (n=180) | Lamwo (n=178) | Pader  (n=177) | Overall (N=714) |  |  |
| **Herd size** |  |  |  |  |  |  |  |
| Less than 3 pigs | 87.2 | 50.0 | 50.6 | 65.0 | 63.2 |  |  |
| Three pigs or more | 12.8 | 50.0 | 49.4 | 35.0 | 36.8 |  |  |
| **Free-roaming pigs** | |  |  |  |  | |  |
| No | 15.6 | 17.8 | 11.2 | 23.2 | 16.9 |  |  |
| Yes | 84.4 | 82.2 | 88.8 | 76.8 | 83.1 |  |  |
| **Borehole water** | |  |  |  |  |  |  |
| No | 9.5 | 5.0 | 13.5 | 4.0 | 8.0 |  |  |
| Yes | 90.5 | 95.0 | 86.5 | 96.0 | 92.0 |  |  |
| **Pig deworming** | |  |  |  |  |  |  |
| No | 77.1 | 62.2 | 71.3 | 71.2 | 70.6 |  |  |
| Yes | 22.9 | 37.8 | 28.7 | 28.8 | 29.4 |  |  |
