# Supplementary material for: Confirmation of a hyperendemic focus of porcine cysticercosis in Northern Uganda: Prevalence and risk factor analysis
Source: PLoS Negl Trop Dis. 2025 Aug 5;19(8):e0013313. doi: 10.1371/journal.pntd.0013313 (PMC12380272; doi:10.1371/journal.pntd.0013313)
Supplement: S5 Table — (DOCX) [file pntd.0013313.s005.docx]

**S5: Table showing the unconditional association of variables with pig seropositivity**

| **Variable/Category** | **Levels** | **Seropositive samples** | **Seronegative samples** | **Total** | **Prev/%** | **Odds ratio** | **P-Value** |
| --- | --- | --- | --- | --- | --- | --- | --- |
| District | Kitgum | 66 | 185 | 251 | 26.3 | 1 (ref) | 0.019 |
|  | Lamwo | 47 | 218 | 265 | 17.7 | 0.54 |  |
|  | Agago | 42 | 228 | 270 | 15.6 | 0.44 |  |
|  | Pader | 49 | 214 | 263 | 18.6 | 0.56 |  |
| Sub-county |  |  |  |  |  |  | 0.02 |
| Respondent | Household head | 119 | 473 | 592 | 20.1 | 1 (ref) | 0.58 |
|  | Other | 85 | 372 | 457 | 18.6 | 0.91 |  |
| Gender | Male | 85 | 372 | 457 | 20.1 | 1 (ref) | 0.58 |
|  | Female | 119 | 473 | 592 | 18.6 | 0.91 |  |
| Education level | Educ 0 | 142 | 623 | 765 | 18.6 | 1 ref | 0.43 |
|  | Educ 1 | 47 | 165 | 212 | 22.2 | 1.24 |  |
|  | Educ 2 | 15 | 57 | 72 | 20.8 | 1.19 |  |
| Age of the respondent | Less than 39 | 120 | 460 | 580 | 20.7 | 1 (ref) | 0.27 |
|  | 39 years or older | 84 | 385 | 469 | 17.9 | 0.84 |  |
| Number of pigs | Less than 3 pigs | 115 | 531 | 646 | 17.8 | 1(ref) | 0.093 |
|  | 3 or more pigs | 89 | 314 | 403 | 22.1 | 1.31 |  |
| Sex of the Pig | Male | 65 | 325 | 390 | 16.7 | 1 (ref) | 0.09 |
|  | Female | 139 | 520 | 659 | 21.1 | 1.34 |  |
| Age of the pig | Less than 8 months | 107 | 559 | 666 | 16.1 | 1 (ref) | 0.0006 |
|  | 8 months or more | 95 | 285 | 380 | 25 | 1.74 |  |
| Breed of the pig | Local | 106 | 528 | 634 | 16.7 | 1 (ref) | 0.006 |
|  | Non-local | 98 | 317 | 415 | 23.6 | 1.53 |  |
| Pig roaming | Total confinement | 25 | 165 | 190 | 13.2 | 1 (ref) | 0.015 |
|  | Any form of free-ranging | 179 | 680 | 859 | 20.8 | 1.74 |  |
| Borehole water | No | 4 | 83 | 87 | 4.6 | 1 (ref) | 0.0005 |
|  | Yes | 200 | 762 | 962 | 26.2 | 5.4 |  |
| Strict com feed | No | 186 | 778 | 964 | 19.3 | 1 (ref) | 0.67 |
|  | Yes | 18 | 67 | 85 | 21.2 | 1.12 |  |
| Food crop residues | No | 34 | 149 | 183 | 18.6 | 1(ref) | 0.84 |
|  | Yes | 170 | 696 | 866 | 19.6 | 1.07 |  |
| Swill | No | 165 | 692 | 857 | 19.3 | 1 (ref) | 0.76 |
|  | Yes | 39 | 153 | 192 | 20.3 | 1.07 |  |
|  |  |  |  |  |  |  |  |
|  |  |  |  |  |  |  |  |
| Home slaughter | No | 154 | 680 | 834 | 18.5 | 1 (ref) | 0.12 |
|  | Yes | 50 | 165 | 215 | 23.3 | 1.34 |  |
| Eat pork out | No | 36 | 173 | 209 | 17.2 | 1 (ref) | 0.38 |
|  | Yes | 168 | 672 | 840 | 20.0 | 1.2 |  |
| Deworming family | Never | 87 | 379 | 466 | 18.7 | 1 (ref) | 0.58 |
|  | Sometimes | 117 | 466 | 583 | 20.1 | 1.09 |  |
| Path toilet | No | 74 | 250 | 324 | 22.8 | 1 (ref) | 0.08 |
|  | Yes | 130 | 593 | 723 | 18.0 | 0.74 |  |
| Toilet in the compound | No | 122 | 556 | 678 | 22.1 | 1 (ref) | 0.07 |
|  | Yes | 82 | 289 | 371 | 18.0 | 0.72 |  |
