# Supplementary material for: Confirmation of a hyperendemic focus of porcine cysticercosis in Northern Uganda: Prevalence and risk factor analysis
Source: PLoS Negl Trop Dis. 2025 Aug 5;19(8):e0013313. doi: 10.1371/journal.pntd.0013313 (PMC12380272; doi:10.1371/journal.pntd.0013313)
Supplement: S6 Table — (DOCX) [file pntd.0013313.s006.docx]

**S6 Table: Results of the univariable analysis of environmental factors**

|  | | | | |
| --- | --- | --- | --- | --- |
| **Variable**  Average rainfall  *Altitude  Slope mean  Land use cover  Mean ndvi  *Distance to the health centre  *Distance to the river  *Distance to main roads | **Estimate Coeff** | **Std. Error** | **z value** | **Pr(>\|z\|)**  0.264  0.115  0.32  0.689  0.121  0.416  0.792  0.15 |
|  | -1.429 | 1.28 | -1.116 |  |
|  | -2.726 | 1.732 | -1.574 |  |
|  | -0.2489 | 0.2502 | -0.995 |  |
|  | -0.03118 | 0.07796 | -0.4 |  |
|  | 1.0412 | 0.6717 | 1.55 |  |
|  | -0.09016 | 0.11081 | -0.814 |  |
