# Supplementary material for: Confirmation of a hyperendemic focus of porcine cysticercosis in Northern Uganda: Prevalence and risk factor analysis
Source: PLoS Negl Trop Dis. 2025 Aug 5;19(8):e0013313. doi: 10.1371/journal.pntd.0013313 (PMC12380272; doi:10.1371/journal.pntd.0013313)
Supplement: S1 Text — (DOCX) [file pntd.0013313.s007.docx]

**S1 Text: Results of K-means clustering analysis**

| **KMEANS clustering analysis**  **Methods**  The Elbow and Gap statistic methods were used to select the optimum number of clusters for Kmeans. The evaluation methods calculated were the total WCSS, between-cluster sum of squares (BCSS), and total sum of squares (TSS) were calculated, Silhouette scores were plotted to visualize clustering quality and Jaccard similarity values from bootstrapping determined cluster reliability.  **Results**   - The optimum number of clusters was found to be two by the two methods. - The mean silhouette width was 0.65 indicating satisfactory clustering. - Cluster 1: Mean village.x = 55.90, Mean cyst_status = 0.1933. and Cluster 2: Mean village.x = 167.48, Mean cyst_status = 0.1907. - Jaccard similarity values were 0.997 for both clusters, indicating excellent stability   The clustering metrics were:   1. Total WCSS: 980,258. 2. BCSS: 3,288,729. 3. Total sum of squares: 4,268,987.   Within-Cluster Sum of Squares (WCSS) is smaller for Cluster 2, indicating that villages in this cluster are more homogeneous compared to Cluster 1 |
| --- |
