# Supplementary figures and images for: Confirmation of a hyperendemic focus of porcine cysticercosis in Northern Uganda: Prevalence and risk factor analysis
Source: PLoS Negl Trop Dis. 2025 Aug 5;19(8):e0013313. doi: 10.1371/journal.pntd.0013313 (PMC12380272; doi:10.1371/journal.pntd.0013313)

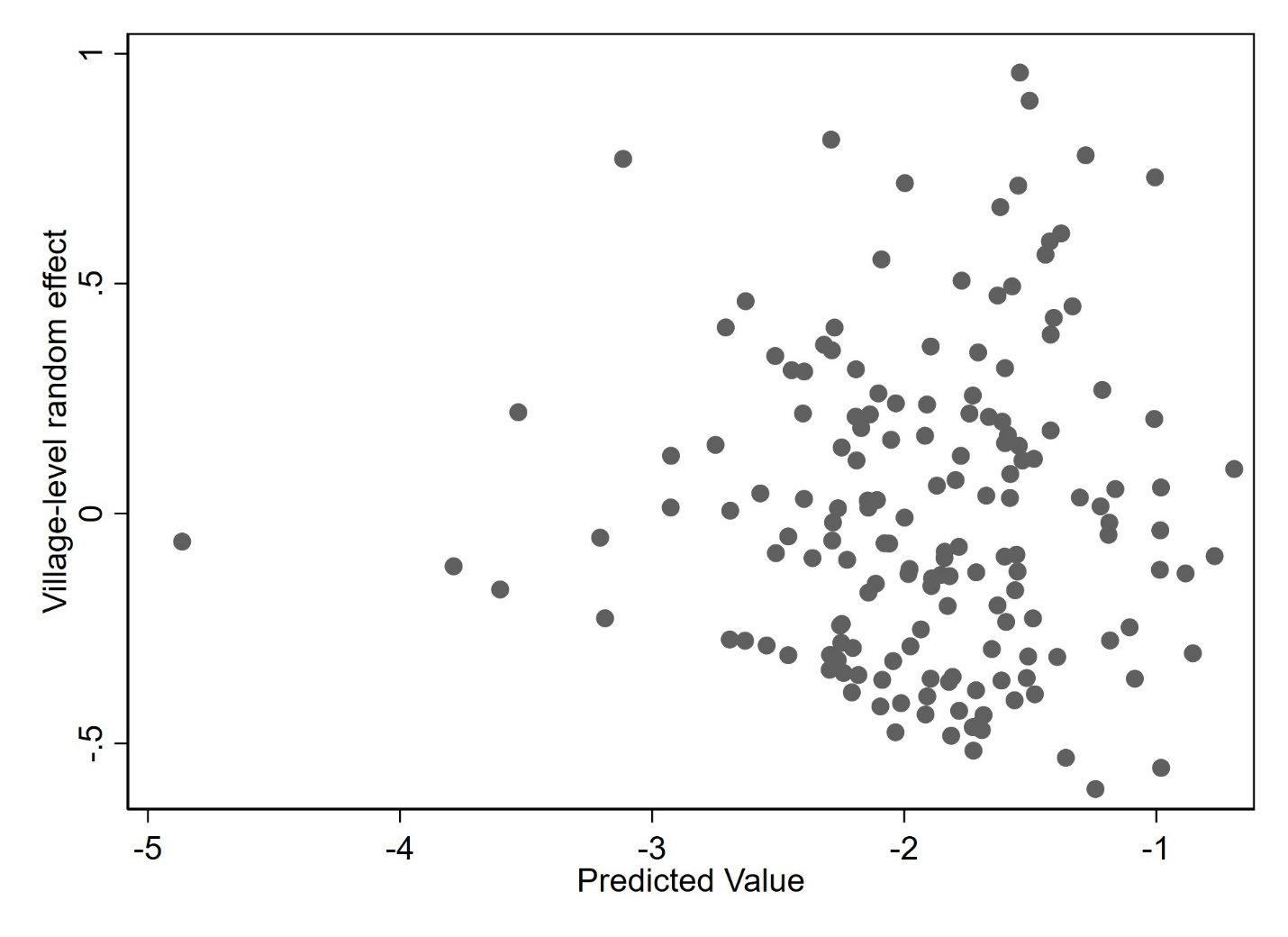

Supplement: S1 Fig — (TIF) [file pntd.0013313.s008.tif]

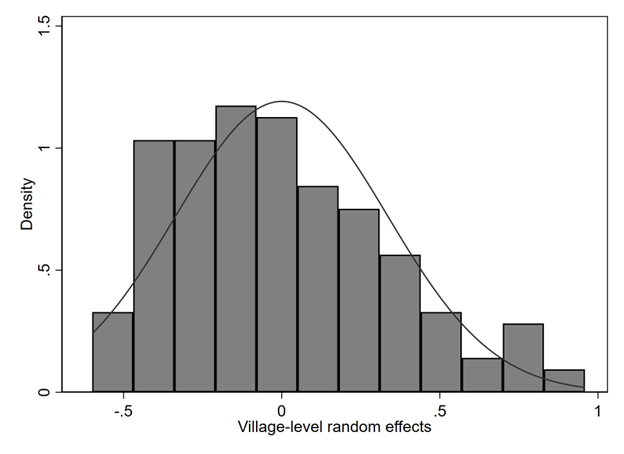

Supplement: S2 Fig — (TIF) [file pntd.0013313.s009.tif]
