# Supplementary material for: Confirmation of a hyperendemic focus of porcine cysticercosis in Northern Uganda: Prevalence and risk factor analysis
Source: PLoS Negl Trop Dis. 2025 Aug 5;19(8):e0013313. doi: 10.1371/journal.pntd.0013313 (PMC12380272; doi:10.1371/journal.pntd.0013313)
Supplement: Attachment — Submitted filename: Review details.docx [file pntd.0013313.s010.docx]

Row 81: add a full stop punctuation after the bibliographical reference

Row 131; row 207-208: row need to be deleted

Row 145: precise the unit used for the population density (per km2 or per sq mi) in the table

Row 145 and Row 155: why did you distribute the number of pigs equitably by district instead of proportionally to be representative? I suggest you talk about this a little in the discussion.

Row 171: Your inclusion criteria is to obtain a pig with a “reasonable body”. Why? You may dismiss some infested pigs that don't have a good body score. And your prevalence might be underestimated. However, your way is also a good reason to detect the importance of the disease in area as you just only choose the best pigs and if you find some positive it’s confirm the result. So, you need more justification for this choice in the methodology or discussion

Row 184 ,row 187 and more others: I don’t understand the reference “S1” … “S2” table

Row 201: (2)????

Row 216: please provide details of the methods of differential diagnosis between T. hydatigena and T. solium as you mention in the results and discussion. Is it just by gross aspects observation or did you use other methods in addition?

Row 226: replace “,” after eg to “:”

Row 228: I don’t understand the direct relationship (represented by the arrow HS and PC 🡪 Infection) between home slaughter and pork consumption with the infection status of the pig

Row 228: please provide the signification of “I” under the legend of the image

Row 287: I noticed some confusion in the sample size calculation and in this result. Is the sampling calculation based on estimate prevalence at animal level or household level? It seems to be based on the prevalence at animal level as your results showed a number of pigs as a sample size. However, in the results, I understood that the ~180 pigs per district became ~180 households. Please clarify or adjust the explanation so as to avoid any potential misinterpretation on the part of the reader.

Row 295: no text related to this table. So, should be moved away or make interpretation accordingly.

Row 299: in my opinion, no statistical difference between female pigs roaming and male pigs roaming

Row 324: should be table 4 instead of 5 (Sanitation, deworming and pork consumption practices)

Row 366 – 368: should be in the methodology

Row 372: should be table 6

Row 386: should be table 7

Row 373 and row 387: I am confused. In the data analysis (row 246) considered significant p value was: P<0.15; why did you still consider some variables that had a p>0.15 (like gender, educational level) in the final model? Are they the household characteristics? If yes, why did you consider them as a potential confounders? And why did you not consider the sex of the pig in the final model?

Row 498: in Uganda
