# Supplementary material for: Confirmation of a hyperendemic focus of porcine cysticercosis in Northern Uganda: Prevalence and risk factor analysis
Source: PLoS Negl Trop Dis. 2025 Aug 5;19(8):e0013313. doi: 10.1371/journal.pntd.0013313 (PMC12380272; doi:10.1371/journal.pntd.0013313)
Supplement: Attachment — Submitted filename: Response to reviewer comments_for submission .docx [file pntd.0013313.s011.docx]

**Response to reviewer comments**

**Dear Editor,**

**Thank you for the constructive feedback and comments from the reviewers. We have comprehensively addressed all the comments and provided a point-by-point response in the section below. We have also highlighted the changes and additions on the manuscript in yellow. A cleaned manuscript has also been attached.**

**We will be happy to provide any further clarifications if needed.**

**Yours sincerely,**

**Lian Thomas**

**Journal Requirements:**

1) We ask that a manuscript source file is provided at Revision. Please upload your manuscript file as a .doc, .docx, .rtf or .tex. If you are providing a .tex file, please upload it under the item type u2018LaTeX Source Fileu2019 and leave your .pdf version as the item type u2018Manuscriptu2019.

2) We do not publish any copyright or trademark symbols that usually accompany proprietary names, eg ©,  ®, or TM  (e.g. next to drug or reagent names). Therefore please remove all instances of trademark/copyright symbols throughout the text, including:
- ® on page: 10 and 11.

3) Please upload all main figures as separate Figure files in .tif or .eps format. For more information about how to convert and format your figure files please see our guidelines: 
<https://journals.plos.org/plosntds/s/figures>

4) We have noticed that you have uploaded Supporting Information files, but you have not included a list of legends. Please add a full list of legends for your Supporting Information files after the references list.

List of legends has been provided at the end reference list.

5) Some material included in your submission may be copyrighted. According to PLOSu2019s copyright policy, authors who use figures or other material (e.g., graphics, clipart, maps) from another author or copyright holder must demonstrate or obtain permission to publish this material under the Creative Commons Attribution 4.0 International (CC BY 4.0) License used by PLOS journals. Please closely review the details of PLOSu2019s copyright requirements here: PLOS Licenses and Copyright. If you need to request permissions from a copyright holder, you may use PLOS's Copyright Content Permission form.
Please respond directly to this email and provide any known details concerning your material's license terms and permissions required for reuse, even if you have not yet obtained copyright permissions or are unsure of your material's copyright compatibility. Once you have responded and addressed all other outstanding technical requirements, you may resubmit your manuscript within Editorial Manager. 
Potential Copyright Issues:

- Figure 2. Please (a) provide a direct link to the base layer of the map (i.e., the country or region border shape) and ensure this is also included in the figure legend; and (b) provide a link to the terms of use / license information for the base layer image or shapefile. We cannot publish proprietary or copyrighted maps (e.g. Google Maps, Mapquest) and the terms of use for your map base layer must be compatible with our CC BY 4.0 license.
Note: if you created the map in a software program like R or ArcGIS, please locate and indicate the source of the basemap shapefile onto which data has been plotted.
If your map was obtained from a copyrighted source please amend the figure so that the base map used is from an openly available source. Alternatively, please provide explicit written permission from the copyright holder granting you the right to publish the material under our CC BY 4.0 license.
If you are unsure whether you can use a map or not, please do reach out and we will be able to help you. The following websites are good examples of where you can source open access or public domain maps:
* U.S. Geological Survey (USGS) - All maps are in the public domain. (<http://www.usgs.gov>)
* PlaniGlobe - All maps are published under a Creative Commons license so please cite u201cPlaniGlobe, <http://www.planiglobe.com>, CC BY 2.0u201d in the image credit after the caption. (<http://www.planiglobe.com/?lang=enl>)
* Natural Earth - All maps are public domain. (<http://www.naturalearthdata.com/about/terms-of-use/>).

Thank you for this observation. The caption has been updated and sources of the base maps indicated and update (line 340 to 345).

6) When completing the data availability statement of the submission form, you indicated that you will make your data available on acceptance. We strongly recommend all authors decide on a data sharing plan before acceptance, as the process can be lengthy and hold up publication timelines. Please note that, though access restrictions are acceptable now, your entire data will need to be made freely accessible if your manuscript is accepted for publication. This policy applies to all data except where public deposition would breach compliance with the protocol approved by your research ethics board. If you are unable to adhere to our open data policy, please kindly revise your statement to explain your reasoning and we will seek the editor's input on an exemption. Please be assured that, once you have provided your new statement, the assessment of your exemption will not hold up the peer review process.

7) Please amend your detailed Financial Disclosure statement. This is published with the article. It must therefore be completed in full sentences and contain the exact wording you wish to be published. Please ensure that the funders and grant numbers match between the Financial Disclosure field and the Funding Information tab in your submission form. Note that the funders must be provided in the same order in both places as well.
- State the initials, alongside each funding source, of each author to receive each grant. For example: "This work was supported by the National Institutes of Health (####### to AM; ###### to CJ) and the National Science Foundation (###### to AM)."
- State what role the funders took in the study. If the funders had no role in your study, please state: "The funders had no role in study design, data collection and analysis, decision to publish, or preparation of the manuscript.".
If you did not receive any funding for this study, please simply state: u201cThe authors received no specific funding for this work.u201d

The funding statement has been updated.

Since the funders did not have a role in the study the statement below has been added.

"The funders had no role in study design, data collection and analysis, decision to publish, or preparation of the manuscript.".

Reviewer #1: The global distribution of Taenia solium is widespread, and taeniasis caused by T. solium is a significant foodborne parasitic disease that has garnered increasing attention in recent years. Conducting epidemiological surveys on taeniasis and cysticercosis in highly endemic regions is of great importance. The author’s investigation on porcine cysticercosis in northern Uganda could provide valuable evidence for understanding the prevalence of T. solium and verify the accuracy of current epidemiological risk assessments in the region. However, the study has several limitations, outlined as follows:

The incidence of taeniasis and cysticercosis exhibits a parallel and concurrent relationship. Therefore, investigations into human infections with adult T. solium (e.g., fecal sampling for egg detection, areca nut-pumpkin seed deworming, and human serological testing) are equally critical. Additionally, the occupational status of participants (e.g., involvement in slaughtering) and dietary habits (e.g., consumption of raw pork or vegetables) are strongly correlated with cysticercosis incidence and should be included in the survey.

Thank you for the comment.

While we appreciate the point raised by the reviewer that taeniasis and cysticercosis exist in parallel, establishing the incidence of taeniasis and human cysticercosis was not the scope of this study due to logistical and funding challenges. We acknowledge these two diseases needed to be studied and we have put them in our plans for future research plans. For examples under a recently funded project in the same study sites we aim to establish the prevalence of human cysticercosis.

Additionally, some of the behaviour mediated drivers of the disease require different approaches and study designs like qualitative studies to understand the behaviour and practices like dietary habits and the drivers as raised by the reviewer. Although, this was not the focus of this study the same study team have reported these aspects elsewhere. For example <https://www.frontiersin.org/journals/veterinary-science/articles/10.3389/fvets.2023.1177526/full> and <https://www.frontiersin.org/journals/veterinary-science/articles/10.3389/fvets.2022.833721/full>.

The points have been alluded to and reference in the introduction line 111 to line 113.

“*Although Uganda ratified WHO’s NTD roadmap 2021-2030, achieving its targets remains unlikely due to: (i) the lack of national and subnational disease-specific control programs, (ii) the absence of an evidence-base confirming hyperendemicity areas for most of the areas in Uganda (iii) a lack of prioritization of T. solium infections control within the national public health and One Health priorities [12] (iv) the lack of understanding of the local contexts that perpetuate the transmission and (v) lack of cross-sectoral coordination at local, regional, and national levels. These problems are not limited to the study area but are national issues that impede efforts to eradicate T. solium alongside other NTDs [12]. Factors perpetuating T. solium transmission such as open defecation, extensive pig production systems, and eating improperly cooked pork are still reported in the area [4,6,14,15].* “

To enhance the representativeness of the findings, the methodology should include not only population density but also details such as the surveyed area’s size, village distribution, and household density.

Thank you for the comment. Some of the data is not available in government sources.

We have updated the table 1 and the text in line 147 to 149 accordingly.

“*Recent population statistics indicate that the average national population human density is 227 persons per sq km, but for the four districts, the average density is 64 people per sq km [18] (Table 1) and much of the area is covered by arable unused land dominated by vegetation and grassland. The total area of the four districts 16,383 square km with Lamwo being the largest district at 5,588 square km (Table 1).”*

Reviewer #2: The methodological approach is generally respected, with the exception of a few specific points, notably on the sampling methodology, which requires more clarification and adjustment according to the results obtained.
This has been addressed throughout the methodology section and in response to the individual comments raised below.
It is important to note that the sample size is large enough to guarantee sufficient statistical power for the study.

The writing is clear and accessible

Thank you for the comments.

**Results**


-

Reviewer #1: The manuscript also contains several issues，The inappropriate presentation of these results may undermine the entire article's quality.:
This comment has not been well understood but if it referred to the comments below, all have been comprehensively addressed.
‌Table 1‌: The unit for "Population density" is unspecified. What do the symbols "HH," "HH*," and "HH" denote?

This have been clarified in the text below the table HH = Household, psq = people per square kilometre and the alphabets in the superscripts denote the source of the data either ^a^National Livestock census report 2021 or ^b^National Housing and Population census 2024. The unit for population density has been given as people per sq km.
‌Line 217‌: The "Data analysis" section lacks a description of statistical software used.

This was been provided with a reference in line 230.

*“Descriptive and summary statistics for all the variables were computed, and p-values determined using Fisher’s exact test [28] in R [29]. The overall and district-level prevalence of T. solium in the districts was determined following adjustment for test specificity (0.947) and sensitivity (0.867) [30]. “*

‌Table 2‌: Are the categorized data (sex, age, reproduction) for pigs presented as proportions or absolute numbers? If proportions, include "%." For example, 270 × 0.433 = 116.91 (non-integer pig counts are illogical). In the "Breed" category, the first group’s data (73.3 + 36.7 = 110) exceed 100%.

Thank you for your comment and highlighting the error on the breed category.

This has been corrected.

In both table the numbers presented are percentages. We made the decision not to include the absolute numbers next to the percentages as this would have made the table clustered.
‌Table 3‌: Similarly, numerical values lack clarification (proportions or counts?).

This is same as above and numbers are percentages. A legend has been provided below the table to highlight this.
‌References‌: Many citations are listed; ensure the referencing style complies with journal guidelines.

The citation style is as per the journal requirements.

Reviewer #2: The result aligns with the analysis plan, except for a few adjustments or modifications that need to be made in the multivariate analysis.

This has been updated as per the comment below on what variables were included in the final model.

Table 3 requires an introductory text for interpretation.

An introductory text to the table has been added in line 320-322 giving a description of the information contained in the table. “*In more than half of the households the household head was interviewed with majority being male (Table 3). Majority of the respondents had primary level of education and below with over half aged below 39 years of age.”*

The table numbers need to be revised.

Thank you for this observation, it was an oversight from our end.

This has been edited accordingly.

Conclusion

Reviewer #2: The subject is original and of great significance form both scientifically and operationally. A concrete example in the discussion part on the proposed One Health approach adapted to their findings would be nice.

This has been added at the conclusion section of the manuscript, line 543 to 547. “*For example, an intervention focusing on the treatment of both humans and pigs with an addition of provision of health education to improve hygiene and husbandry practices*.”

Reviewer #2: The study is grounded in a clearly defined research question and addresses a methodological gap in assessing the prevalence of porcine cysticercosis, which serves as a proxy for the evaluation and control of neurocysticercosis.
I have only a few comments, attached in this review, to help improve the manuscript prior to publication

Row 81: add a full stop punctuation after the bibliographical reference.

This has been added.

Row 131; row 207-208: row need to be deleted

This comment is not understood but the lines have been checked and any issues corrected.

Row 145: precise the unit used for the population density (per km2 or per sq mi) in the table

This has been updated on the table

Row 145 and Row 155: why did you distribute the number of pigs equitably by district instead of proportionally to be representative? I suggest you talk about this a little in the discussion.

Thank you for this comment.

At the time of carrying out sample size calculation we only had pig population numbers for 2008 which we deemed inaccurate because the northern Uganda region was coming out of conflict and pig farming grew significantly since then to the date of planning this study. The National Livestock census of 2021 was published later after the study was completed. We therefore assumed an equal distribution across districts with an infinite population for the sample size.

Row 171: Your inclusion criteria is to obtain a pig with a “reasonable body”. Why? You may dismiss some infested pigs that don't have a good body score. And your prevalence might be underestimated. However, your way is also a good reason to detect the importance of the disease in area as you just only choose the best pigs and if you find some positive it’s confirm the result. So, you need more justification for this choice in the methodology or discussion

Our main reasoning not to sample emaciated, pregnant or lactating sows pigs due to welfare considerations and avoid stressing the animal especially the lactating and pregnant ones . However, this has been edited to show that we avoided physically emaciated pigs to reduce the risk of complications during sampling.

Row 184 ,row 187 and more others: I don’t understand the reference “S1” … “S2” table

This refers to tables in the supplementary materials and is named based on the instructions to the author from the journal.

Row 201: (2)????

This was an error and has been corrected by inserting the correct reference.

Row 216: please provide details of the methods of differential diagnosis between T. hydatigena and T. solium as you mention in the results and discussion. Is it just by gross aspects observation or did you use other methods in addition?

Thank you for this comment.

Yes this was just gross observation and we have clarified this in the methods, (Line 226-229), but unfortunately, we did not carry out PCR to speciate.

*“T. hydatigena were identified on gross pathology as large (>1cm) thin-walled fluid filled cysts with long slender necks at the attachment with liver capsule or other peritoneal surfaces rather than within the musculature*.”

Row 226: replace “,” after eg to “:”

This has been corrected.

Row 228: I don’t understand the direct relationship (represented by the arrow HS and PC 🡪 Infection) between home slaughter and pork consumption with the infection status of the pig

Thank you for this comment.

The relationship here is, people who slaughter pigs at home and consume pork are more likely to consume the infective cysts because in most cases pigs slaughtered at home are mostly not inspected. Even though it appears not to act directly on the outcome of interest and to act through an intervening variable like toilet use, it remains the important exposure variable. Intervening variables are normally excluded from the model.

Row 228: please provide the signification of “I” under the legend of the image

The ‘I’ denotes the outcome of interest. This has been clarified below the figure (line 235 to line 239).

Row 287: I noticed some confusion in the sample size calculation and in this result. Is the sampling calculation based on estimate prevalence at animal level or household level? It seems to be based on the prevalence at animal level as your results showed a number of pigs as a sample size. However, in the results, I understood that the ~180 pigs per district became ~180 households. Please clarify or adjust the explanation so as to avoid any potential misinterpretation on the part of the reader.

The sample size was calculated at two levels. First animal level to be able to estimate the prevalence and secondly at the household level to be able to determine the risk factors.

This has been clarified in the methods section under sample size calculation line 156 to line 169.

“*The sample size for determining the prevalence of PCC was calculated using the formula n = [Z^2^P(1-P)]/d^2^ as defined by Thrusfield [19], where n is the required sample size; Z is the multiplier from a standard normal distribution (1.96) at a probability level of 0.05; P is the estimate of prevalence taken at 13.6 % as reported by Alarakol et al [20] in Amuru and Gulu which border the districts under study; and d is the desired precision level (0.05). From the calculation, a sample size of 180 pigs per district was required for the study making a total of 720 pigs. We sampled 720 households from which a maximum of two eligible pigs were sampled. We confirmed that this sample size was sufficient for a risk factor analysis. We calculated the sample size for risk factor analysis using the formulae for 2-independent sample binomial comparison [21]. Absence of a latrine used as the risk factor of interest for this calculation with the prevalence of PCC in households with latrines assumed to be (12.7%) and those without (2.2%) as reported in Kungu et al., [22]. The target sample size to be able to find at least 95 households without toilets was 380 households based on 25% toilet coverage in the study districts indicating that our initial sample size was sufficient for risk factor analysis.* “

Row 295: no text related to this table. So, should be moved away or make interpretation accordingly.

An interpretation for Table 3 has been added and the table referenced line 318-320

“*In more than half of the households the household head was interviewed with majority being male (Table 3). Majority of the respondents had primary level of education and below with over half aged below 39 years of age.”*

Row 299: in my opinion, no statistical difference between female pigs roaming and male pigs roaming

We agree the line has been deleted.

Row 324: should be table 4 instead of 5 (Sanitation, deworming and pork consumption practices)

All the table numbers have been updated. This was an error from our editing.

Row 366 – 368: should be in the methodology

The section on development of the model has been moved to the methodology section lines 299 to 310.

Row 372: should be table 6

All the table numbers have been updated. This was an error from our editing.

Row 386: should be table 7

All the table numbers have been updated. This was an error from our editing.

Row 373 and row 387: I am confused. In the data analysis (row 246) considered significant p value was: P<0.15; why did you still consider some variables that had a p>0.15 (like gender, educational level) in the final model? Are they the household characteristics? If yes, why did you consider them as a potential confounders? And why did you not consider the sex of the pig in the final model?

Thank you for this comment. In the causal diagram Figure 1, household characteristics were confounders and were therefore forced into the model even when the p was greater than 0.15 at univariable level. On the other hand, sex of the pig is an intervening variable and therefore not included in the final model. This has been clarified in the manuscript (line 391 to 394).

“*Variables with p-values below 0.15, variables that were components of interaction terms of specific interest, and household characteristics deemed to be potential confounders were retained for multivariable analysis. Pig level characteristics were intervening variables and were excluded from the model*.”

Row 498: in Uganda

This is noted and has been corrected.
